# A negative fluid balance may compromise nutritional delivery in mechanically ventilated critically ill children: a retrospective observational cohort study

**DOI:** 10.1186/s13054-026-05951-9

**Published:** 2026-03-19

**Authors:** Melany Gaetani, Marina Santschi, David Anthony, Haifa Mtaweh

**Affiliations:** 1 Department of Critical Care Medicine, Hospital for Sick Children, 555 University Ave, Toronto, M5G18X ON Canada; 2 Institute of Health Policy, Management and Evaluation, University of Toronto, Toronto, Canada; 3 Department of Paediatrics, University of Toronto, Toronto, Canada; 4 Information Services, The Hospital for Sick Children, Toronto, ON Canada

**Keywords:** Paediatric critical care, Fluid balance, Nutrition, Mechanical ventilation, Energy delivery

## Abstract

**Background:**

Nutrition and fluid balance are core components of supportive care in paediatric critical illness, yet their interaction remains poorly defined. We examined the relationship between fluid balance trajectories and nutritional delivery during the first week of paediatric intensive care.

**Methods:**

We performed a retrospective cohort study of mechanically ventilated children (0–18 years) admitted for ≥ 24 h to a tertiary PICU between 2019 and 2024 who received furosemide. Patients with diagnoses requiring disease-specific fluid strategies were excluded. Fluid balance was calculated in 12-hour intervals and cumulatively. Nutritional delivery from enteral and parenteral sources was expressed as a percentage of predicted energy expenditure (DPEE). The primary outcome was cumulative nutritional delivery at ICU discharge or day 7, comparing patients with negative versus neutral/positive cumulative fluid balance. Multivariable linear regression adjusted for age, illness severity, vasoactive support, mechanical ventilation duration, and ICU length of stay.

**Results:**

Among 511 included patients (median age 42 months), nutritional support increased progressively, with > 75% receiving nutrition by 72 h and > 90% by day 7; enteral feeding predominated. Fluid balance was initially positive, transitioning toward neutrality and negative balance after approximately 72 h. As cumulative fluid balance declined, cumulative nutritional delivery increased. In adjusted analyses, negative cumulative fluid balance was independently associated with a 50% reduction in delivered energy compared with neutral or positive balance (*p* = 0.002). Younger age was associated with lower nutritional delivery, while longer ICU stay was associated with modest increases.

**Conclusions:**

During early paediatric critical illness, fluid balance and nutritional delivery follow interdependent trajectories. Strategies aimed at achieving negative fluid balance are associated with substantially reduced energy delivery, independent of illness severity. These findings identify fluid and nutrition management as linked, modifiable targets and support integrated approaches to optimize supportive care in critically ill children.

**Supplementary Information:**

The online version contains supplementary material available at 10.1186/s13054-026-05951-9.

## Introduction

Energy is the currency of life—and in critical illness, demand often outpaces supply. Acute stress responses increase energy expenditure and protein catabolism, leading to rapid lean muscle mass loss. Inadequate caloric and protein intake worsens this catabolic state, contributing to muscle wasting and functional decline [1–4]. Adequate nutritional support is therefore a central component of paediatric critical care management.

Nutrition, however, also contributes to daily fluid intake, and its delivery occurs within the broader context of fluid management [5]. This relationship is particularly relevant in the paediatric intensive care unit (PICU), where children often receive large volumes of fluid for resuscitation, vasoactive infusions, medications, blood products, and maintenance requirements [6]. These competing fluid demands can limit the remaining fluid “space” available for nutritional support [7].

Fluid balance is an important determinant of outcomes in critically ill patients. Excessive fluid accumulation has been linked to prolonged ventilation, acute kidney injury, poor wound healing, and increased mortality [8–11], and it can obscure true weight or muscle mass loss, complicating nutritional assessment [12]. In mechanically ventilated patients, achieving a neutral or negative fluid balance may improve oxygenation, facilitate ventilator weaning, and support recovery of organ function [13, 14]. However, strategies aimed at avoiding fluid overload may inadvertently restrict the volume available for nutrition delivery.

The tension between fluid management and nutritional adequacy is a common challenge in the PICU. Although enteral nutrition is physiologically preferred [15, 16], fewer than half of children in PICUs meet their nutritional targets [17, 18]. This shortfall is frequently due to gastrointestinal dysfunction [17, 19–22], interruptions for procedures [16, 20, 21, 23], lower caloric density of enteral feeds compared to parenteral options [24, 25], and fluid restriction intended to avoid overload [16, 22, 23, 26–28].

Despite the recognized importance of fluid balance, paediatric studies have largely evaluated prescribed fluid restriction rather actual fluid balance when examining its relationship with nutrition delivery. Many studies define restriction using thresholds such as < 80% of Holliday-Segar formula, but this may not reflect real-time fluid status or its nutritional consequences [23, 26, 28, 29]. Accordingly, this study explores the relationship between fluid balance and nutritional intake in a non-cardiac, medical and surgical PICU population, with the hypothesis that nutritional deficits may persist even after neutral or negative fluid balance is achieved.

## Methods

### Study design, setting and population of interest

We performed a retrospective cohort study in a 44-bed combined Paediatric and Cardiac Critical Care Unit at The Hospital for Sick Children (SickKids), in Toronto, Canada. Eligible patients were critically ill children between the ages of 0–18 years, admitted to the unit between April 1, 2019 to June 30, 2024. We included patients admitted to the intensive care unit for 24 h or more, mechanically ventilated, and had received one or more doses of furosemide therapy, defined as the provision of one or more of the following formulations of furosemide: intravenous injection, intravenous infusion, or enteral. Patients with diagnoses requiring specific fluid therapy such as rhabdomyolysis, dysnatremias, burns, sickle cell disease, tumor lysis syndrome, hyperglycemic crises/diabetic ketoacidosis, stroke, hyperammonemia/inborn errors of metabolism and congenital heart disease were excluded. This study was approved by the SickKids institutional research ethics board (1000081323) and procedures to conduct this study were followed in accordance with the ethical standards of the Helsinki Declaration of 1975. All reporting was performed in accordance with the Strengthening the Reporting of Observational Studies in Epidemiology (STROBE) guidelines [30].

### Data acquisition and management

Demographic information and clinical characteristics were abstracted from the electronic medical record (EMR) into a secure database. Abstracted clinical data included ICU admission and discharge date and time to calculate length of ICU stay, the volume of fluid administered and removed each hour in the ICU to calculate the 12 hourly fluid balance in ml, medications, laboratory values, ventilatory time, nutritional intake including enteral and parenteral sources, duration of invasive mechanical ventilation, and mortality. We additionally collected data to allow the calculation of the paediatric index of mortality 3 score [31] and vasoactive-inotropic Score (VIS) scores [32]. Fluid balances were separated a priori into 12 hourly intervals to reflect real-world clinical practice of fluid evaluation at the study centre. For subgroup analysis, patients were considered primarily parenterally fed if they received TPN and lipids for more than 50% of the observation period (> 3.5days).

**Fig. 1:**
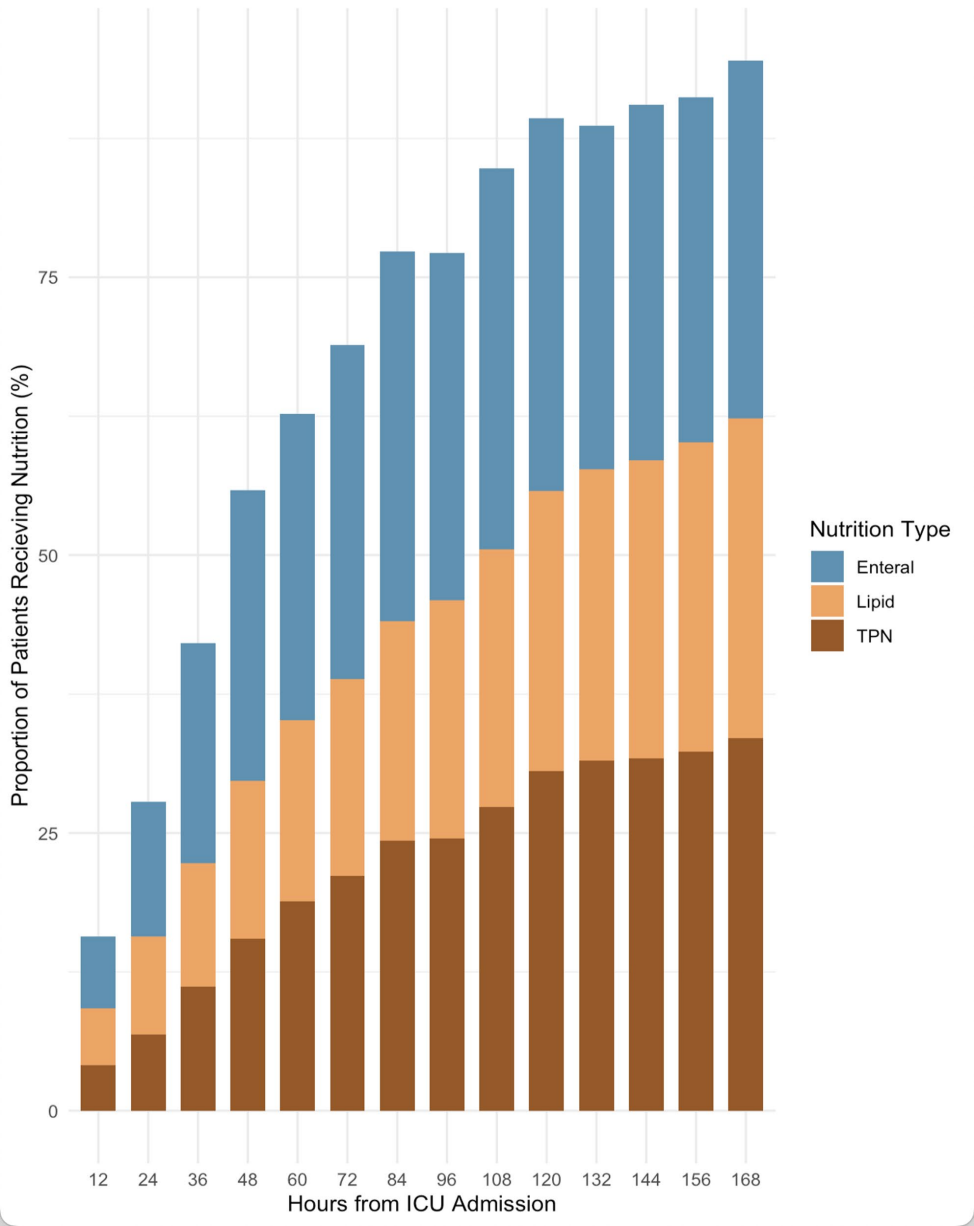
Nutrition Delivery in ICU. outlines the proportion of patients in this study receiving different nutritional interventions. The most common type of nutritional delivery was enteral across all intervals. By day 5 of ICU admission more than three quarters of patients were receiving nutritional support

### Outcomes

The primary outcome was the cumulative nutritional delivery at discharge or in the first 7 days of ICU comparing patients who had a negative fluid balance to those with a positive or neutral fluid balance. The primary outcome was represented as the percentage of energy delivered from enteral and parenteral nutrition (excluding non-TPN intravenous dextrose) to predicted energy expenditure using the Schofield equation (DPEE) [33].

### Analysis

Demographic, clinical characteristics, and outcomes were described using frequencies and percentages (categorical variables), means and standard deviations (SD; continuous and normally distributed variables), or medians and interquartile ranges (IQR; continuous and skewed variables). Wilcoxon rank sum tests were used to compare medians and Fisher’s exact test was used for comparisons of categorical data. Linear regression model was used for univariate evaluation of the cumulative DPEE as the dependent variable and age, sex, diagnostic category, vasoactive inotropic score, illness severity, length of mechanical ventilation as independent variables. Variables with p-value < 0.2 were included in the multivariate linear regression. Due to right-skewness and non-normal distribution of the outcome variable, a natural log transformation was applied to outcome variable (percentage of total delivered energy expenditure) to satisfy model assumptions of normality, linearity, and homoscedasticity. Patients with missing data for any included variable were excluded from the analysis. Model assumptions were evaluated using standard diagnostic plots, including residuals vs. fitted values, normal Q-Q plots, scale-location plots, and residuals vs. leverage plots. Variance inflation factors (VIFs) were assessed to evaluate multicollinearity with a threshold of < 5 considered acceptable for inclusion in the final model. Results are presented as beta coefficients (β) with corresponding 95% confidence intervals (CI) and p-values. To aid clinical interpretation, exponentiated coefficients were reported as relative percent changes in the original outcome scale, calculated as 100 × (exp(β) − 1). The Wilcoxon Signed rank test was performed to investigate the difference between fluid intake and negative fluid balance on individual 12 h ICU intervals. The resulting p values were corrected for the multiple test situations with the Bonferroni formula.

All statistical analyses were conducted using R version 4.1.0 (The R Foundation for Statistical Computing, Vienna, Austria). A two-sided p-value < 0.05 was considered statistically significant.

**Fig. 2:**
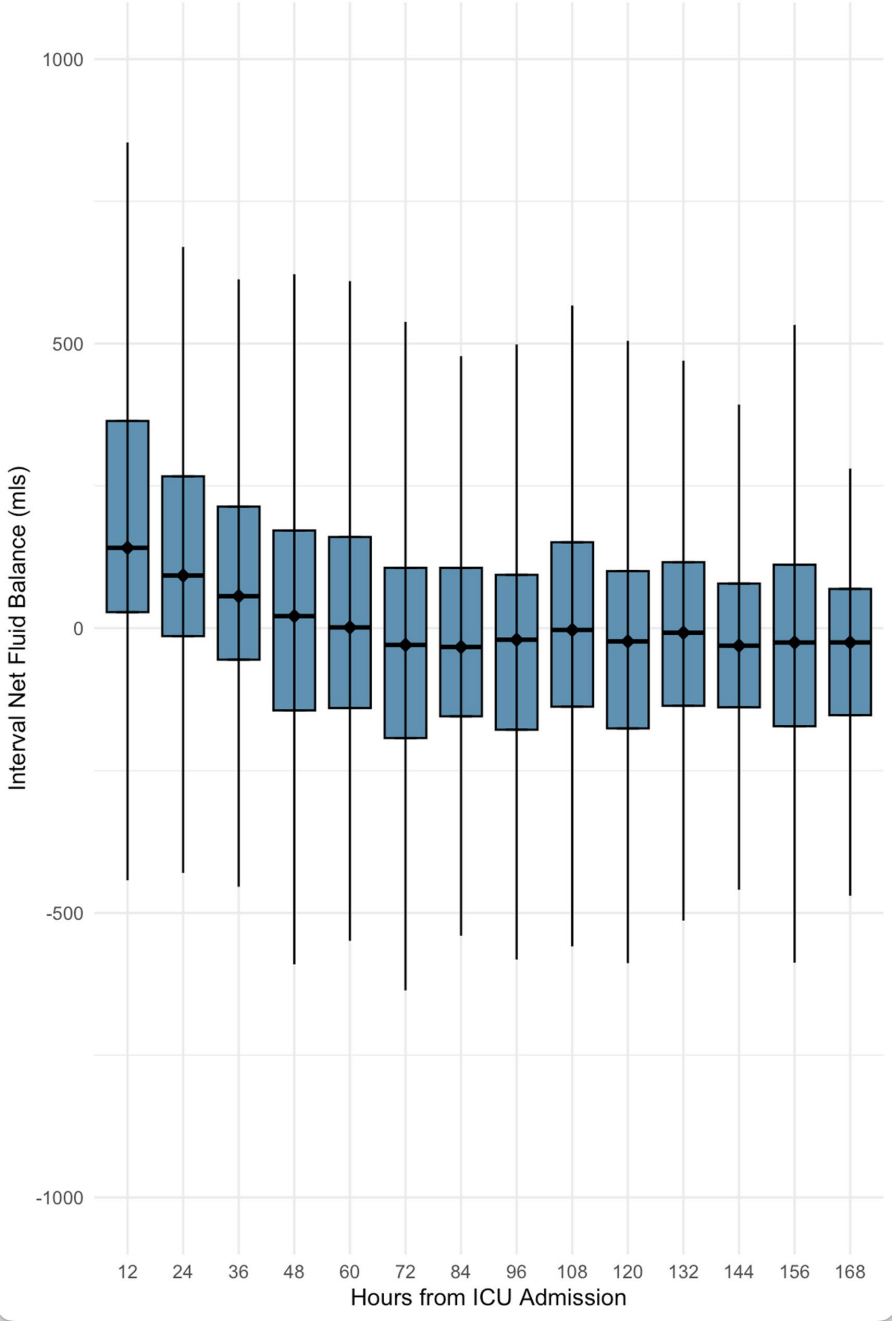
Fluid Balance by Intervals. The net fluid balance median and interquartile range is represented for each 12 h interval in ICU. The net fluid balance decreases in each interval until approximately 3 days in ICU where it remains more stable

## Results

A total of 4293 patients were admitted to the paediatric intensive care unit between April 1, 2019 and June 30, 2024. Of these, 511 were admitted for greater than 24 h, invasively mechanically ventilated, and administered furosemide (Supplementary Fig. 1). The median age was 42 months [IQR: 5, 137], and the median weight was 12 kg [IQR: 5.7, 30]. Males comprised 54.8% of the cohort. When stratified by fluid balance status, those with a negative cumulative fluid balance (*n* = 207) were significantly younger (median 19 vs. 56 months, *p* < 0.001) and had lower median body weight (7.46 kg vs. 14.20 kg, *p* < 0.001) than those with neutral or positive fluid balance. ICU length of stay and mechanical ventilation (MV) time were longer among those with negative fluid balance, although only MV time reached statistical significance (median 116.57 h vs. 86.56 h, *p* = 0.001). Diagnostic categories were similar between groups (Table 1).

**Table 1:** Patient Characteristics

Variable	Overall (*n* = 511)	Neutral/Positive Fluid Balance (*n* = 246)	Negative Fluid Balance (*n* = 207)	*p*-value
Male sex, n (%)	280 (54.8%)	133 (54.1%)	122 (58.9%)	0.344
Weight (kg), median [IQR]	12.20 [5.73, 30.00]	14.20 [8.17, 30.53]	7.46 [4.80, 23.30]	< 0.001
Age (months), median [IQR]	42.00 [5.00, 137.00]	56.00 [14.25, 143.75]	19.00 [2.00, 122.00]	< 0.001
OD, median [IQR]	98.24 [93.04, 99.44]	97.86 [86.60, 99.26]	98.90 [95.03, 99.79]	< 0.001
VIS, median [IQR]	13.65 [8.00, 22.75]	14.50 [8.00, 27.26]	14.00 [8.00, 20.00]	0.466
ICU LOS (h), median [IQR]	188.23 [98.20, 382.79]	190.76 [92.92, 385.90]	204.00 [118.76, 409.65]	0.096
Diagnostic Category, n (%)				0.825
Cardiac Arrest	14 (2.7%)	7 (2.8%)	5 (2.4%)	
Cardiac Diagnosis	6 (1.2%)	2 (0.8%)	2 (1.0%)	
Hematology/Oncology	4 (0.8%)	2 (0.8%)	1 (0.5%)	
Neurological	71 (13.9%)	37 (15.0%)	23 (11.1%)	
Post-operative non cardiac	42 (8.2%)	22 (8.9%)	17 (8.2%)	
Respiratory	248 (48.5%)	112 (45.5%)	110 (53.1%)	
Sepsis	47 (9.2%)	23 (9.3%)	20 (9.7%)	
Solid Organ Transplant	13 (2.5%)	9 (3.7%)	3 (1.4%)	
Trauma	27 (5.3%)	12 (4.9%)	10 (4.8%)	
Other	39 (7.6%)	20 (8.1%)	16 (7.7%)	
MV time (h), median [IQR]	100.18 [42.14, 219.71]	86.56 [36.50, 210.59]	116.57 [67.38, 242.41]	0.001
ICU death, n (%)	44 (8.6)	18 (7.3)	17 (8.2)	0.722

Table 1 represents baseline characteristics of the study population overall and stratified by fluid balance. Continuous variables are reported as median [interquartile range (IQR)] and were compared using nonparametric tests due to non-normal distribution. Categorical variables are reported as countsand percentages and were compared using the chi-square test. The negative fluid balance group had significantly lower age and weight, and longer durations of mechanical ventilation, compared to patients without a negative balance. No significant differences were observed in ICU mortality, VIS scores, or diagnostic categories between the groups

OD: Odds of death (derived from PIM3); VIS: Vasoactive inotropic score; ICU: intensive care unit; LOS: length of stay; MV: mechanical ventilation

### Nutrition delivery

The proportion of patients receiving any form of nutrition increased steadily from ICU admission to day 7 (168 h). By 72 h, over 75% of patients had received at least one form of nutrition, increasing to over 90% by 168 h. Enteral nutrition was the most common modality, increasing from < 10% at 12 h to approximately 88% by day 7. TPN use increased from 5% at 12 h to just over 35% at 168 h, while lipid supplementation rose from 7% to approximately 65% over the same period. These data reflect a progressive, multimodal approach to nutritional support, with increasing reliance on enteral routes over time (Fig. 1).

The relationship between medication volume and nutritional delivery was also explored. In unadjusted analysis, cumulative IV medication volume was positively associated with cumulative nutrition delivery (β = 0.485 kcal per mL, *p* < 0.001; R² = 0.028). After adjustment for ICU length of stay, IV medication volume remained independently associated with cumulative nutrition (β = 0.267 kcal per mL, *p* < 0.001), while ICU length of stay was the dominant predictor (β = 8.26 kcal per unit increase in LOS, *p* < 0.001; t = 55.9). The large t value for ICU length of stay reflects the expected time-dependent accumulation of nutrition delivery, indicating that duration of ICU stay accounts for the majority of variability in cumulative energy received (model R² = 0.388).

**Fig. 3:**
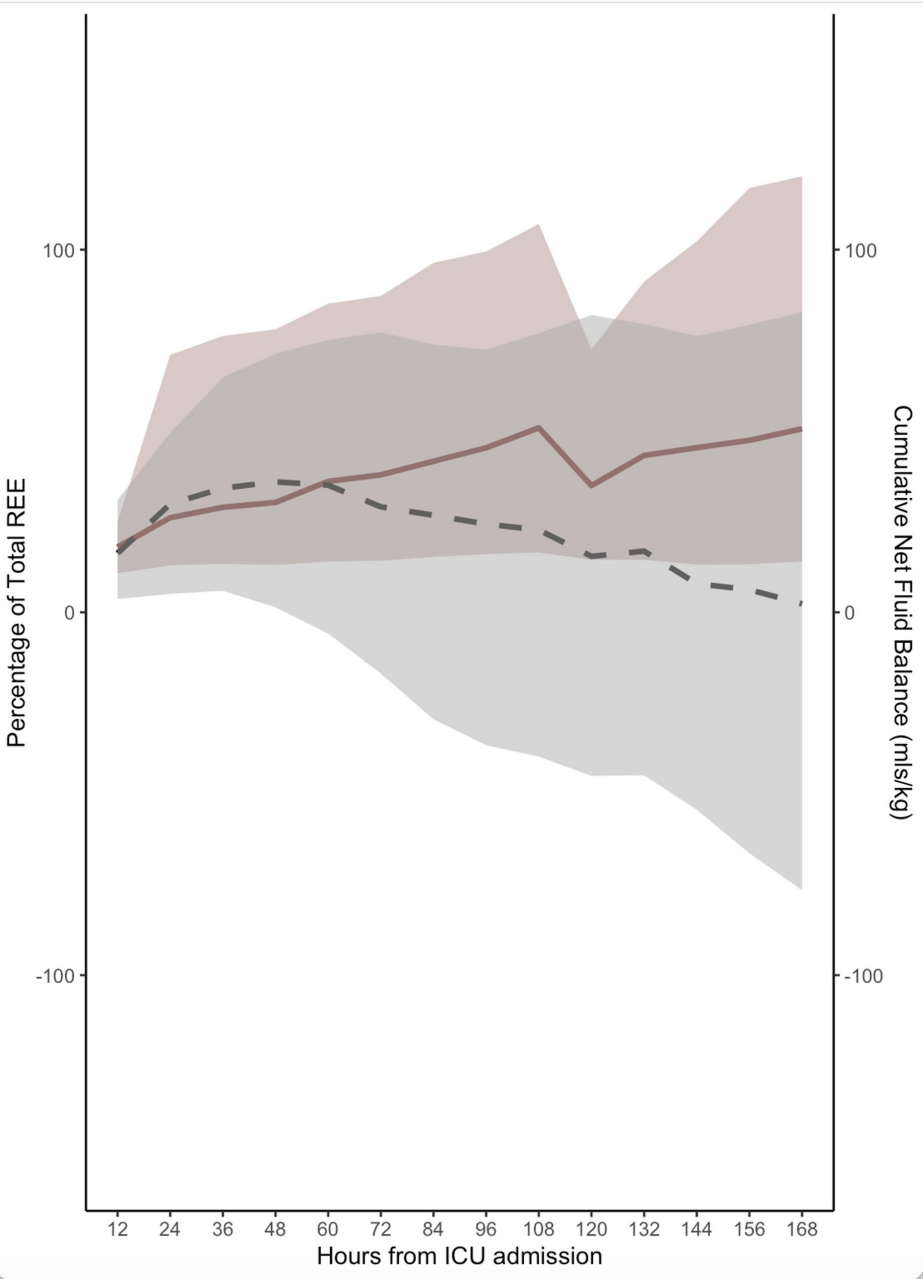
Energy Delivery and Net Cumulative Fluid Balance. shows the intersection of cumulative net fluid balance and the percentage of resting energy expenditure delivered to patients. The dashed line and corresponding grey banner represent the median and interquartile range of cumulative net fluid balance in ICU over a 7 day period. The red line and corresponding red banner represent the median and interquartile range of the delivered percentage of total resting energy expenditure. REE = resting energy expenditure

## Fluid balance by interval and cumulatively

The interval net fluid balance decreased progressively during the first week of ICU admission. Early time intervals were characterized by net positive balances, with the highest median occurring in the first 24 h (+ 170 mL, IQR: 29.9 to 454 mL). This declined steadily over time, becoming more neutral by approximately 60 h with a median + 4.4 mL, IQR: − 145 to 174 mL in the fifth interval. By 72 h onward, interval balances became consistently net negative, with medians ranging from approximately − 26 to − 38 mL and lower IQR bounds exceeding − 200 mL in some intervals (Fig. 2).

Cumulative net fluid balance and nutritional delivery demonstrated opposing trends over time in the first week of ICU admission. Cumulative fluid balance (CB) initially rose, with a median of 16.3 mL/kg [IQR: 3.7, 31.0 mL/kg] at the first interval (12–24 h), peaking at 36.0 mL/kg [IQR: 1.4, 71.3 mL/kg] by the fourth interval. After this point, cumulative balance began to decline progressively, dropping to 24.3 mL/kg [IQR: − 36.6, 72.4 mL/kg] by day 4 and further down to just 2.3 mL/kg [IQR: − 76.6, 82.8 mL/kg] by the end of the first week. In contrast, cumulative nutritional delivery steadily increased. The percentage of total delivered calories to predicted energy expenditure (EE) increased from a median of 18.0% [IQR: 10.8, 24.9%] at 12 h to 30.3% [IQR: 13.1, 78.1%] by the fourth interval, and reached 50.6% [IQR: 14.1, 120.0%] by the final interval. Shaded confidence bands around these trajectories suggest considerable interpatient variability (Fig. 3). As shown in Supplementary Fig. 2, patients with negative fluid balance received lower median hourly fluid intake across multiple intervals, indicating that fluid balance differences were at least partially attributable to differences in administered intake in addition to fluid removal.

## Negative fluid balance and nutritional delivery

In univariate analysis, patients with a negative cumulative fluid balance at day 7 or ICU discharge received a lower percentage of their estimated energy requirements compared to those with neutral or positive balances. The median DPEE was 23.4% [IQR: 9.7%, 85.9%] in the negative balance group versus 34.9% [IQR: 16.2%, 87.3%] in the non-negative group. However, this difference was not statistically significant in linear regression. Negative fluid balance was associated with a non-significant 10.1% decrease in cumulative nutrition delivery (β = -10.10, *p* = 0.588).

**Fig. 4:**
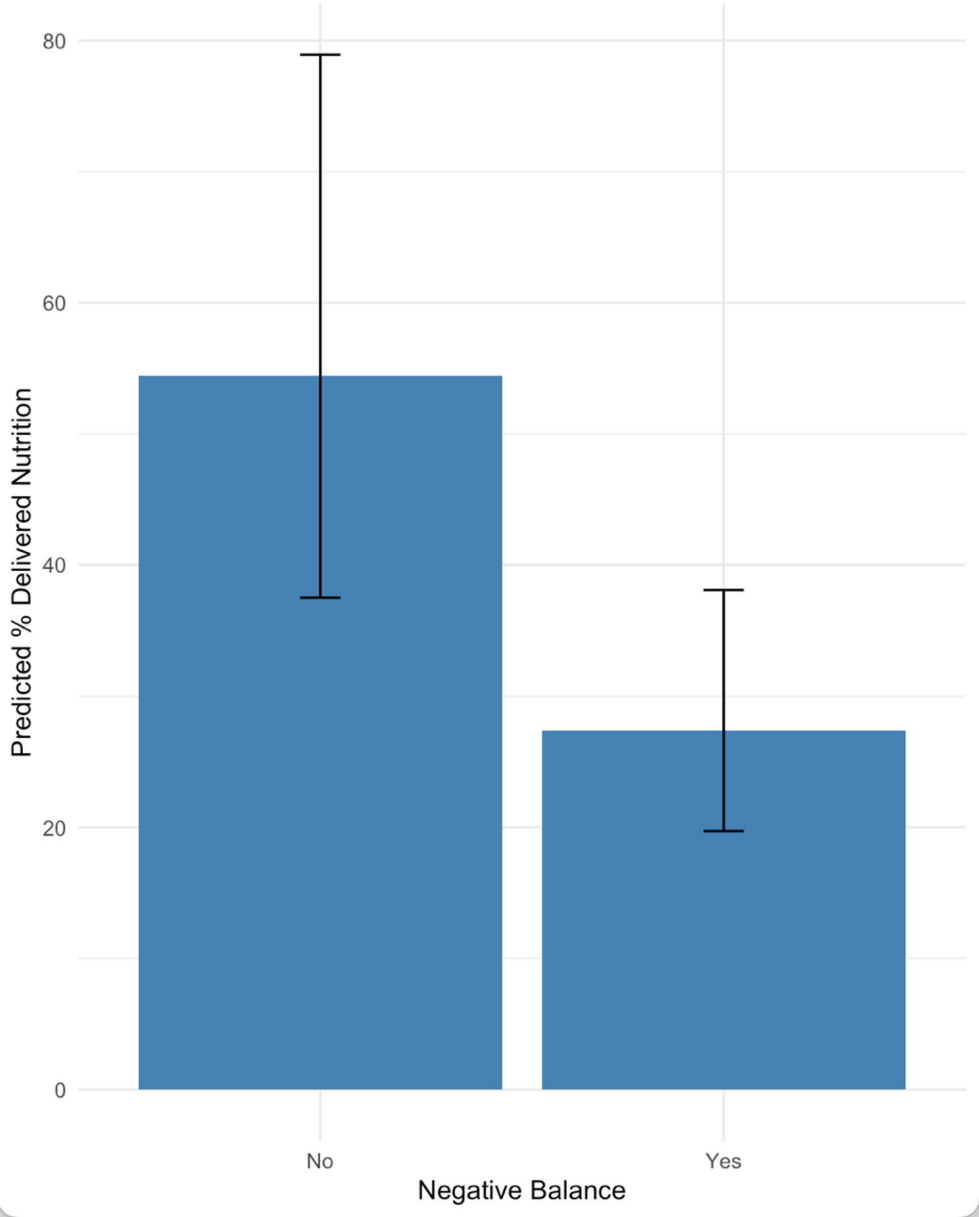
Negative Fluid Balance and Nutritional Delivery. shows the model-predicted percent of cumulative delivery by negative fluid balance status. Predicted values were derived from a linear regression model with a log-transformed outcome, adjusting for illness severity-based odds of death (PIM3), vasoactive-inotropic score (VIS), patient age, ICU length of stay and mechanical ventilation time. Blue bars represent predicted percent delivered for patients with and without a negative fluid balance, based on typical values for other covariates (medians). Black lines indicate 95% confidence intervals around the predicted means. Patients with a negative fluid balance had a significantly lower predicted percent delivered, approximately 50% lower, compared to those without a negative balance

In the adjusted analyses, negative fluid balance was independently associated with a 50% reduction in nutritional delivery (β = -0.686, 95% CI: -1.118 to -0.254; *p* = 0.002). Additionally, younger age was associated with lower nutritional intake (-0.6% per month; *p* < 0.001), and longer ICU stay was associated with a slight increase in intake (+ 0.1% per day; *p* = 0.030). No association was detected between vasoactive inotrope score or mechanical ventilation time and DPEE (Supplementary Table 1). Patients with a negative cumulative fluid balance received significantly less nutrition than those without. The mean DPEE was approximately 32% in the negative balance group compared to 51% in the neutral/positive balance group (difference of approximately 19% points). The 95% confidence intervals for each group showed minimal overlap (Negative: 20–37%; Positive: 37–79%) (Fig. 4).

During the 7-day observation period, 74 of 511 patients (14%) received parenteral nutrition for more than 50% of the study period, with a median exposure of 80% (IQR 71–95%) of the observation period. Among these patients who predominantly received parenteral nutrition, negative fluid balance was not associated with cumulative nutrition delivery (β = 14.35, *p* = 0.55) in the univariate model. However, in adjusted analyses, negative fluid balance was independently associated with a 32% increase in cumulative parenteral nutrition delivery (β = 0.28, *p* = 0.01). In patients who received primarily enterally nutrition, negative fluid balance was associated with a non-significant decrease in cumulative nutrition delivery in unadjusted analyses (β = −10.10, *p* = 0.588), while adjusted analyses demonstrated an independent association between negative fluid balance and a 50% reduction in nutritional delivery (β = −0.686, 95% CI − 1.118 to − 0.254; *p* = 0.002).

## Discussion

In this large retrospective study of 511 critically ill children, we identified a clear and consistent association between fluid balance and nutritional intake during the first week of ICU admission. These findings highlight three clinically relevant considerations: how nutrition is delivered to mechanically ventilated children, how fluid balance evolves and is prioritized over time, and how these two components of supportive care interact in practice. Together, our results demonstrate that nutritional delivery and fluid balance are closely linked and must be considered concurrently when caring for critically ill paediatric patients.

Nutritional support was initiated early in mechanically ventilated children and increased progressively over the first week of ICU admission, with more than 75% of patients receiving some form of nutrition by 72 h and over 90% by day 7. Enteral nutrition was the predominant modality throughout, supplemented by parenteral nutrition and intravenous lipid delivery. Despite this high rate of nutritional exposure, the proportion of estimated energy requirements achieved was variable, particularly during the early phase of illness when fluid accumulation was greatest. This shortfall is clinically meaningful as inadequate nutrition has been associated with impaired wound healing, immune dysfunction, postoperative pneumonia, and multiorgan failure in adults [34–37], as well as higher rates of discharge home rather than rehabilitation among adults receiving adequate energy delivery [38]. In paediatric critical care, malnutrition has similarly been linked to increased morbidity and mortality [3, 4, 7, 39, 40], and early enteral nutrition to reduced risk-adjusted mortality and shorter length of stay [41].

Fluid balance management evolved in parallel with nutritional delivery. Children start their ICU stay with net-positive fluid balance associated with resuscitation and stabilization, during which nutritional delivery remained limited, followed by a transition toward fluid neutrality or negative balance as nutrition increased and resuscitative priorities resolved. Because both enteral and parenteral feeding contribute to daily fluid intake, strategies aimed at restricting or removing fluid may inadvertently constrain nutritional advancement. Positive fluid balance may further impair gastrointestinal function through intestinal wall edema, delayed gastric emptying, and ileus, presenting a physiologic barrier to enteral tolerance [42].

These interactions between fluid status and nutrition are particularly relevant in the context of mechanical ventilation. In our cohort, most children were ventilated during the period of greatest fluid accumulation, when inadequate energy delivery and impaired gastrointestinal tolerance may contribute to loss of lean body mass, respiratory muscle weakness, and delayed liberation from ventilation. Similar relationships have been observed in neonatal populations, where higher fluid intake is associated with increased risk of bronchopulmonary dysplasia [43], and inadequate energy provision during early postnatal life has been associated with disrupted lung development [44, 45]. Together, these findings suggest that fluid balance, nutrition, and respiratory recovery are mechanistically interconnected.

Children achieving a negative cumulative fluid balance received substantially less nutrition than those with neutral or positive balances, even after adjustment for age, illness severity, ICU length of stay, vasoactive support, and duration of mechanical ventilation. Negative balance was independently associated with an approximately 50% reduction in delivered energy relative to estimated targets, confirming that nutritional goals and fluid balance targets are interdependent and not achieved independently in clinical practice. Similar challenges have been reported in other paediatric populations, including children with congenital heart disease and neonates requiring extracorporeal support, where fluid restriction reduces the likelihood of meeting energy and protein goals [23, 26, 27], and this challenge is echoed in international PICU practice surveys [46–48].

Importantly, the association between negative fluid balance and reduced nutritional delivery differed according to the route of nutrition in our cohort. Among patients receiving predominantly parenteral nutrition, negative fluid balance was not associated with reduced nutritional intake in unadjusted analyses and was independently associated with increased cumulative nutrition delivery after adjustment. In contrast, among patients receiving primarily enteral nutrition, negative fluid balance was independently associated with a substantial reduction in delivered nutrition. These findings suggest that the observed relationship between fluid balance and nutrition is largely driven by the fluid-dependent nature of enteral feeding. Enteral nutrition typically requires larger fluid volumes to meet caloric targets, whereas parenteral formulations can be concentrated to provide higher caloric density within smaller volumes. As a result, strategies aimed at achieving negative fluid balance may disproportionately limit enteral nutrition delivery while having less impact on parenteral nutrition administration. This finding has not been reported in the adult or paediatric literature previously.

We also observed an association between intravenous medication volume and nutrition delivery. The relationship was modest and largely explained by ICU length of stay, which was the dominant predictor of cumulative nutritional intake. This likely reflects the time-dependent nature of both therapies, children who remain in the ICU longer receive greater cumulative medication exposure and greater cumulative nutrition. Nevertheless, this finding highlights the broader fluid context within which nutrition is delivered in the PICU. Intravenous medications contribute substantially to daily fluid intake through drug diluents, carrier infusions, and continuous vasoactive therapies as reported previously in the literature [49, 50]. These medication related fluids occupy part of the limited daily fluid allowance and may reduce the volume available for nutritional support, particularly when restrictive fluid strategies are employed. The analysis of hourly fluid intake further supported this relationship. Patients who achieved negative fluid balance received consistently lower median hourly fluid intake than those who remained in positive balance, indicating that fluid balance differences were attributable not only to active fluid removal but also to differences in administered intake as has been previously reported [51–53]. This pattern suggests that clinical decisions aimed at achieving fluid neutrality or negative balance often involve reductions in administered fluids, which may inadvertently constrain nutritional delivery.

This study has several limitations. First, as a single-centre retrospective cohort study, exposure timing and treatment decisions could not be controlled, and residual confounding remains possible despite adjustment for age, illness severity, vasoactive support, mechanical ventilation duration, and ICU length of stay. Second, we studied a population receiving invasive mechanical ventilation and loop diuretics, reflecting a subset of critically ill children with fluid shifts and diuretic exposure; these findings may not generalize to children who are spontaneously breathing or not receiving fluid restriction. Third, nutritional adequacy was assessed using predicted energy expenditure and did not include protein delivery, micronutrient provision, or cumulative caloric deficit beyond seven days. Fourth, data on gastrointestinal intolerance, was not collected, limiting mechanistic insight into enteral feeding limitations. Finally, we did not evaluate longer-term clinical outcomes such as functional recovery, rates of extubation failure, or post-discharge growth, which would strengthen the clinical relevance of this study.

The strengths of the findings in this study suggest that optimizing outcomes in critically ill children requires intentional integration of nutrition and fluid management strategies. Advancing enteral nutrition as early as feasible, minimizing unnecessary fluid exposure, and anticipating periods where fluid management goals may constrain nutritional adequacy may help mitigate cumulative nutritional deficits. Developing coordinated protocols that prioritize both adequate early energy delivery and judicious fluid management represents a key opportunity to improve outcomes in paediatric critical care.

## Conclusion

This study is the first and largest to clearly demonstrate a significant association between fluid balance and nutritional delivery during the early course of invasively ventilated, critically ill, children. Our findings provide insight into potentially modifiable aspects of care, highlighting opportunities to improve energy delivery through coordinated approaches to nutrition and fluid management, with the goal of optimizing patient outcomes. Future work should focus on strategies that support earlier and sustained enteral nutrition while achieving optimal fluid balance. Ultimately, advancing paediatric critical care will require a shift toward integrated supportive care models that recognize nutrition and fluid balance as interdependent, rather than competing, therapeutic priorities.

## Supplementary Information

Supplementary Material 1

## Data Availability

Available upon reasonable request.
