# Supplementary material for: A negative fluid balance may compromise nutritional delivery in mechanically ventilated critically ill children: a retrospective observational cohort study
Source: Crit Care. 2026 Mar 19;30:206. doi: 10.1186/s13054-026-05951-9 (PMC13123147; doi:10.1186/s13054-026-05951-9)
Supplement: Supplementary file 1 — Supplementary Material 1 [file 13054_2026_5951_MOESM2_ESM.docx]

**Supplementary Figure 1: Patient Flowchart**

**643 patients excluded due to diagnoses with specific fluid therapy requirements such as**
rhabdomyolysis, dysnatremias, burns, sickle cell disease, tumor lysis syndrome***,*** hyperglycemic crises/diabetic ketoacidosis, stroke, hyperammonemia/inborn errors of metabolism.

**4,293** patient admissions to the Paediatric Critical Care Unit in which patients received intravenous fluids

**3,650** patient admissions following exclusion for fluid therapy

**1092** received one or more doses of furosemide

**991** had an ICU LOS > 24 hours

**511** invasively ventilated and included in the study

Study cohort selection for invasively ventilated pediatric patients receiving furosemide admitted more than 24 hours in the ICU. *ICU LOS = Intensive care unit length of stay.*

**Supplementary Table 1**

| **Predictor** | **Coefficient (β)** | **95% CI** | **p-value** | **% Change in Outcome (exp(β) - 1)** |
| --- | --- | --- | --- | --- |
| Negative fluid balance | -0.686 | (-1.118, -0.254) | 0.002 ** | −50% |
| Odds of Death (PIM3) | -0.004 | (-0.012, 0.004) | 0.299 | −0.4% |
| Vasoactive Inotropic Score | 0.0037 | (-0.0026, 0.0100) | 0.251 | 0.40% |
| Age, months | -0.0061 | (-0.0090, -0.0031) | <0.001 ** | −0.6% per month |
| ICU length of stay, hours | 0.0011 | (0.0001, 0.0020) | 0.030 * | +0.1% per day |
| Mechanical Ventilation Time, hours | -0.00033 | (-0.0016, 0.00096) | 0.62 | −0.03% |

Multivariable linear regression model predicting the natural log of cumulative percent delivered. Coefficients (β) represent the estimated change in the log-transformed outcome for a one-unit increase in each predictor, adjusting for all other variables in the model. Percent change in the original scale of delivered nutrition is derived by exponentiating the coefficients (exp(β) − 1). Negative fluid balance, younger patient age, and longer ICU length of stay were significantly associated with changes in cumulative delivery percentage. The model was adjusted for odds of death, vasoactive-inotropic score, mechanical ventilation time and included 197 observations. Statistical significance was defined as p < 0.05.

**
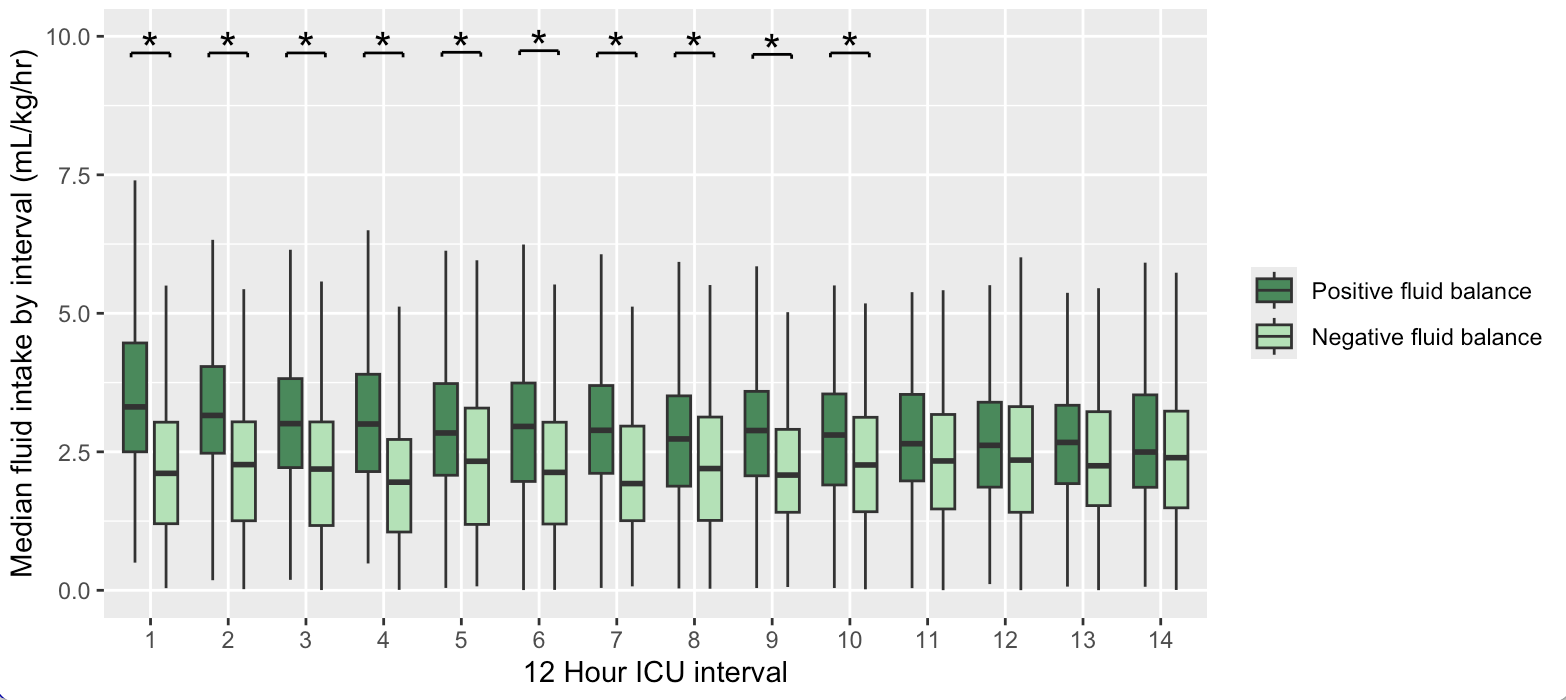
Supplementary Figure 2: Median Hourly Fluid Intake by 12-Hour ICU Interval Stratified by Fluid Balance Status**

Boxplots display the patient-level median hourly fluid intake (mL/kg/hr) within each 12-hour ICU interval (intervals 1–14), stratified by fluid balance status. Dark green represents patients with positive fluid balance and light green represents patients with negative fluid balance. For each interval, the median intake was calculated per patient and compared between groups using the Wilcoxon rank-sum test. P-values were adjusted for multiple comparisons using the Bonferroni method. Asterisks denote statistically significant differences between groups (p < 0.05). Boxes represent the interquartile range (IQR), horizontal lines indicate medians, and whiskers extend to 1.5× IQR.
